# Lovastatin Alleviates α-Synuclein Aggregation and Phosphorylation in Cellular Models of Synucleinopathy

**DOI:** 10.3389/fnmol.2021.682320

**Published:** 2021-07-26

**Authors:** Lijun Dai, Jiannan Wang, Mingyang He, Min Xiong, Ye Tian, Chaoyang Liu, Zhentao Zhang

**Affiliations:** ^1^Department of Neurology, Renmin Hospital of Wuhan University, Wuhan, China; ^2^Hubei Provincial Institute for Food Supervision and Test, Wuhan, China; ^3^Research Center for Environment and Health, Zhongnan University of Economics and Law, Wuhan, China

**Keywords:** Parkinson’s disease, protein aggregation, α-synuclein, statins, casein kinase 2, haistone acetylation

## Abstract

Parkinson’s disease (PD) is one of the most common neurodegenerative diseases. Pathologically, it is characterized by the aberrant aggregation of α-synuclein (α-syn) in neurons. Clinical evidence shows that patients with hypercholesterolemia are more likely to get PD, while lovastatin users have a lower risk of suffering from it. In this study, we investigated the effects of lovastatin on the aggregation and phosphorylation of α-syn *in vitro*. Our results demonstrate that α-syn preformed fibrils induce the phosphorylation and aggregation of α-syn in HEK293 cells stably transfected with α-syn-GFP and SH-SY5Y cells as well, which could be attenuated by in a concentration-dependent manner. Besides, lovastatin inhibited oxidative stress, histone acetylation, and the activation of casein kinase 2 (CK2). Collectively, lovastatin alleviates α-syn aggregation and phosphorylation in cellular models of synucleinopathy, indicating its potential value of being adopted in the management of PD.

## Introduction

Parkinson’s disease (PD) is the second most common neurodegenerative disorder after Alzheimer’s disease (Armstrong and Okun, 2020). Epidemiological studies have shown that approximately 1% of seniors older than 60 were involved in PD (Feigin et al., 2017). It was pathologically characterized by the degeneration of dopaminergic neurons in the substantia nigra pars compacta (SNpc) and the formation of Lewy bodies (LBs), of which the major protein component is the α-synuclein (α-syn). Although the pathogenesis of PD remains to be elucidated, the α-syn toxicity hypothesis is the most widely accepted. It states that α-syn could be hyperphosphorylated and aggregated under some obscure pathological conditions to form toxic oligomers and fibrils, which lead to neuronal impairment (Visanji et al., 2019). Injection of α-syn pre-formed fibers (PFFs) or pathological aggregates extracted from the brains of PD patients triggers the development of PD-like pathology and behavioral impairments in wild-type mice, which is consistent with the prion hypothesis of PD that the propagation of pathological α-syn plays a pivotal role in the initiation of PD (Luk et al., 2012a, b).

α-Syn is subjected to sophisticated post-transcriptional modifications (PTMs), which significantly alter its intrinsic properties of aggregation and neurotoxicity. Most of them (90%) are phosphorylated at the S129 site in the brains of PD patients. On the contrary, only a small fraction (<4%) is phosphorylated in the controls (Fujiwara et al., 2002). The phosphorylation of α-syn at S129 regulates physiological function and remarkably promotes their aggregation, critically participates in the pathogenesis of PD as an important molecular event. Casein kinase 2 (CK2) is the major one of the kinases that mediate α-syn phosphorylation at S129 (Perez et al., 2011; Wu et al., 2020). It consists of two regulatory subunits β and two catalytic subunits α that mainly contribute to the catalytic activity.

Currently, only a few approved drugs are utilized to partly relieve the motor symptoms of PD. Unfortunately, none of them was proved to stop or reverse this inevitable disease progression. Several clinical studies have shown that statins, the commonly-used lipid-lowering drugs could reduce the risk of PD (Lin et al., 2016; Swallow et al., 2016; Brakedal et al., 2017; Kawada, 2017; Carroll et al., 2019). A double−blind randomized placebo−controlled clinical trial found that lovastatin treatment was associated with a slower exacerbation of motor symptoms in PD patients (Lin et al., 2021). In addition, statin administration prevents the onset of PD in patients with diabetes (Lin et al., 2016). The use of another statin, simvastatin, is also relevant to a decreased risk of PD (Kawada, 2017). These results indicate that statins are beneficial for reducing the risk of PD, but also slow down its rate of progression. However, the underlying mechanisms mediating the protective effects of lovastatin remain unclear. Here we characterized the effects of lovastatin on α-synucleinopathy and found that lovastatin reduced α-syn aggregation and phosphorylation. Besides, it also attenuated oxidative stress and epigenetic dysregulation in these cellular models.

## Materials and Methods

### Regents

Lovastatin and Thioflavin T were purchased from Sigma (St. Louis, MO, United States). Lipofectamine-2000, β-D-thiogalactoside (IPTG), and Alexa Fluor 488/594-conjugated secondary antibodies were purchased from Thermo Fisher Scientific (Waltham, MA, United States). Anti-p-S129 and anti-GAPDH were purchased from BioLegend (San Diego, CA, United States). Anti-CK2α, anti-CK2β, anti-Histone 3, and anti-Histone 4 were purchased from Proteintech (Wuhan, China). Anti-AcH3K9 and AcH4K5 were purchased from Cell Signaling Technology (Danvers, MA, United States). CK2 and GRK5 kits were purchased from Jianglaibio (Shanghai, China).

### Cell Culture

HEK293 cells and SH-SY5Y cells were cultured in DMEM medium containing 10% (100 mg/ml) fetal bovine serum, 100 units/mL of penicillin, and 100 units/mL of streptomycin in a humidified atmosphere containing 5% CO_2_ at 37°C. The HEK293 cell line stably expressing α-syn-GFP (α-syn-GFP-HEK293 cells) was established according to the methods described before (Holmes et al., 2014; Kaufman et al., 2016; Deshayes et al., 2019). Briefly, the HEK293 cells were infected with lentivirus harboring human α-syn cDNA with a GFP-tag to the C-terminal. The cells were exposed to puromycin for selective screening. Cells were passed every 3–4 days at a 1:4 ratio. The medium was replaced every 2 days. Exogenous preformed α-syn fibers (PFFs) were transfected to induced α-syn aggregates.

### Purification of Recombinant Human α-Syn

The expression and purification of recombinant human α-syn were performed as previously described (Volpicelli-Daley et al., 2014). The human α-syn coding sequences with a 6xHis-tag were cloned into the PRK172 plasmid and transduced into the BL21 *Escherichia coli* strain. Cells were cultured in Luria-Bertani broth medium with 100 mg/mL ampicillin at 37°C. 0.6 mM isopropyl IPTG was added until the OD600 reaches about 0.6, followed by 6-h additional incubation for α-syn expression. Then the protein was purified using 6xHis-affinity chromatography and lyophilized according to the method described before (Volpicelli-Daley et al., 2014). The purity of the recombinant α-syn was verified by Coomassie blue staining. The protein concentration was determined using bicinchoninic acid (BCA) assay (Thermo Fisher Scientific).

### Preparation of α-Syn PFFs

To prepare α-syn PFFs, the lyophilized protein was dissolved in PBS and centrifuged at 100,000 rpm for 1 h at 4°C. The protein concentration in the supernatant was determined, and diluted to 1 mg/ml. Then the solution was incubated on a thermostatic mixer at 37°C, rotated at 1,000 rpm for 5–7 days for obtaining PFFs as described previously (Volpicelli-Daley et al., 2014; Kim et al., 2019). PFFs solution was assessed by Thioflavin-T (ThT) assay. Fibrils were sonicated on ice (Sonics Vibra cell) with a fine tip for 20 s, 20% amplitude, pulse 1 s on/1 s off. Sonicated α-syn fibrils were aliquoted, snap frozen in liquid nitrogen, and stored at −80°C.

### Induction of α-Syn Aggregation in α-syn-GFP-HEK293 Cells

For transduction of α-syn PFFs, the α-syn-GFP-HEK293 cells were cultured in 24-well plates at a density of 20,000 cells per well. Equal volumes of Lipofectamine 2000 (Thermo Fisher Scientific) and α-syn PFFs solutions, were diluted in Opti-MEM medium and mixed to a final concentration of 2.8% (v/v), followed by a 20-min incubation before adding into cell medium. 48 h later, the level of phosphorylated α-syn and inclusions was determined by immunofluorescence and Western blot.

### Lovastatin Treatment

Lovastatin (Sigma, St. Louis, MO, United States) was dissolved in DMSO to prepare a stock solution of 6 mM. Cells were treated with different concentrations of lovastatin for 4 h and then transduced with α-syn PFFs. Lovastatin was maintained in the medium. The cells were harvested 48 h after transfection.

### Extraction of Soluble and Insoluble α-Syn

For sequential extraction of soluble and insoluble α-syn, the cells were washed twice with PBS and scraped into 1% Triton X-100 (v/v)/Tris-buffered saline (TBS) (50 mM Tris, 150 mM NaCl, pH 7.6) containing protease and phosphatase inhibitors (Sigma-Aldrich, Switzerland). After sonication [0.5-s pulse at an amplitude of 20%, ten times (Ningbo Toshiba Ultrasonic Cell Crusher JY99-IIDN, China)], lysates were incubated on ice for 30 min and centrifuged at 100,000 × *g* for 30 min at 4°C. The supernatant (soluble fraction) was collected, while the pellet was washed in 1% Triton X-100/TBS, sonicated, and centrifuged for 30 min at 100,000 × *g*. The supernatant was discarded and the pellet (insoluble fraction) was dissolved in 2% sodium dodecyl sulfate (SDS)/TBS supplemented with protease inhibitor cocktail and phosphatase inhibitor, sonicated (0.5-s pulse at an amplitude of 20%, 15 times), and incubated at room temperature for 30 min. After centrifugation, the supernatant containing insoluble fractions was retained.

### Western Blot

Cells were washed twice with PBS and scraped into ice-cold NP40 cell lysis buffer containing protease and phosphatase inhibitors. The lysates were centrifuged at 15,000 rpm for 20 min at 4°C. The protein concentration was determined using the BCA assay. Proteins were separated on SDS/15% polyacrylamide gels, transferred onto nitrocellulose membranes (Thermo Fisher Scientific, Switzerland) utilizing a semi-dry system (Bio-Rad, Switzerland). The membranes were blocked in 5% non-fat milk (50 mg/ml) in TBST, and incubated with the following primary antibodies: anti-p-S129 antibody (Proteintech, 1:1,000, United States), anti-GAPDH antibody (BioLegend, 1:1,000, United States), anti-CK2α antibody (Proteintech, 1:1,000, United States), anti-CK2β antibody (Proteintech, 1:1,000, United States), anti-AcH3K9 antibody (Cell Signaling Technology, 1:1,000, United States), anti-AcH4K5 antibody (Cell Signaling Technology, 1:1,000, United States), anti-Histone 3 antibody (Proteintech, 1:1,000), and anti-Histone 4 antibody (Proteintech, 1:1,000) overnight at 4°C. The membranes were washed three times in TBST and labeled with horseradish peroxidase (HRP)-conjugated secondary antibodies. The signals were developed by enhanced chemiluminescent (ECL). ImageJ software was used to measure the intensity of Western blot bands as described previously (Jensen, 2013).

### Immunofluorescence

For immunofluorescence, cells were fixed and permeabilized with 4% paraformaldehyde (40 mg/ml) supplemented with 1% TX-100 (v/v) for 15 min, washed three times in PBS, blocked using 3% BSA (30 mg/ml) at room temperature for 30 min, and then incubated with the primary antibody as follows: anti-p-S129 antibody (1:1,000), anti-CK2α antibody (1:100), anti-CK2β antibody (1:100), anti-p-S129 antibody (1:500), anti-ACH3K9 antibody (1:500), and anti-ACH4K5 antibody (1:500) overnight at 4°C. Then, the cells were incubated with Alexa Fluor 488/594 anti-mouse/Rabbit secondary antibody (1:500) for 2 h at room temperature shield from light. The slides were stained with DAPI (300 nM) for 30 s, washed three times in PBS, mounted, and imaged under an Olympus inverted fluorescence microscope (Olympus TH4-200, Japan). For detection of α-syn aggregates, the α-syn-GFP-HEK293 cells were treated with α-syn PFFs for 48 h, and then fixed and permeabilized with 4% paraformaldehyde (40 mg/ml) supplemented with 1% TX-100 (v/v) for 15 min. The number of aggregates was observed under a 40-fold objective lens (Olympus TH4-200, Japan). At least 120 cells in more than three visual fields were involved for statistical analysis. For 3D imaging, slices were scanned along Z-stack and rebuild using a Laser Scanning Confocal Microscopy (Leica, Germany). ImageJ software was used to calculate the number of aggregates and fluorescence intensity.

### Cell Viability Assay

Cell viability was performed using the Cell Counting Kit-8 viability assay (CCK-8, Dojindo, Kumamoto, Japan). Briefly, cells were plated at a density of 1 × 10^4^ cells/100 μl in 96-well plates and exposed to α-syn PFFs. After 48-h treatment, 10 μl of CCK8 solution was added to each well and incubated at 37°C for 2 h. The medium was removed and washed twice with PBS. Absorbance at 450 nm was measured using SpectraMax Plus 384 Microplate Reader. Cell viability was expressed as the percentage versus the control group. The experiment was independently repeated three times.

### Determination of Intracellular ROS, Lipid Peroxidation, and Hydrogen Peroxide

Determination of intracellular reactive oxygen species (ROS), lipid peroxidation, and hydrogen peroxide levels was performed using DCFH-DA assay kit, malondialdehyde (MDA) assay kits, and hydrogen peroxide assay kits respectively (all from Jiancheng Bioengineering Institute, Nanjing, China) according to the manufacturer’s instructions. All results were normalized to the corresponding total protein content.

### Statistical Analyses

All data were expressed as means ± SEM. Statistical analyses were performed using one-way ANOVA followed by Tukey’s *post hoc* multiple comparisons using Prism GraphPad 8 (GraphPad Software Inc., San Diego, CA, United States). *P* < 0.05 was considered statistically significant. All experiments were performed in triplicate for at least three independent trials.

## Results

### Lovastatin Inhibits α-Syn Aggregation in α-Syn-GFP-HEK293 Cells

**FIGURE 1:**
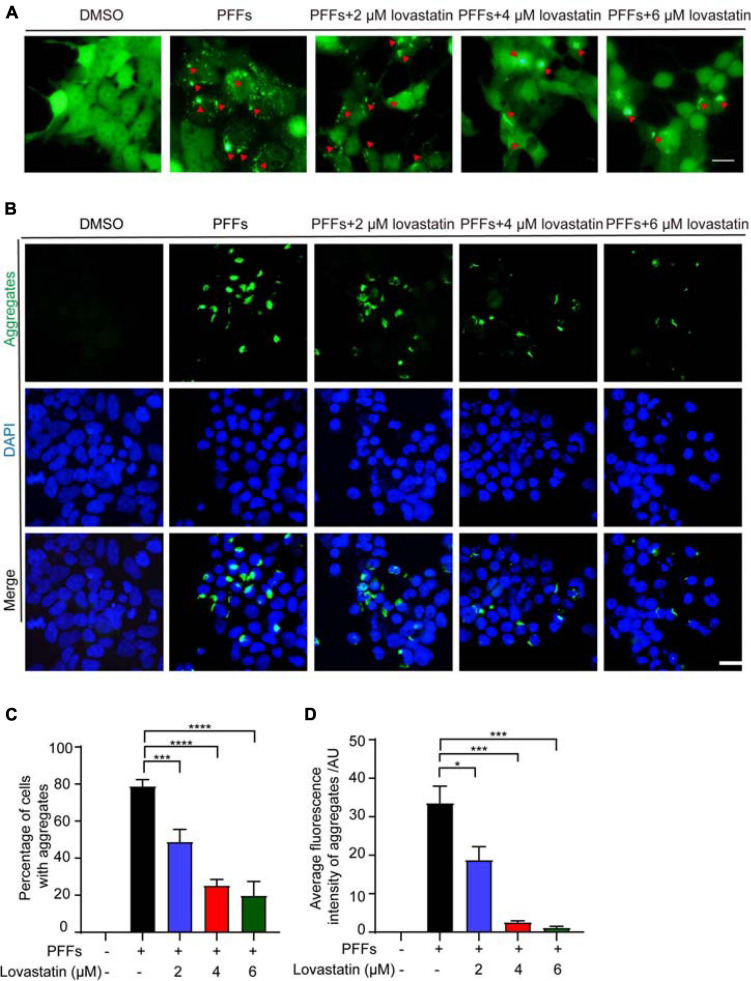
Lovastatin inhibits α-syn aggregation in GFP-α-syn-HEK293 cells. **(A)** Intracellular α-syn aggregates assay. Green aggregates indicate α-syn aggregates. The aggregates in the lovastatin group were significantly reduced compared with the α-syn-PFFs group. **(B)** Insoluble α-syn aggregates after permeabilization. Compared with the α-syn PFFs group, the insoluble α-syn aggregates (green) was significantly reduced in the lovastatin group. **(C)** Counting of α-syn aggregates. At least 120 cells from more than three fields were counted in the experiments. **(D)** Quantification of the insoluble α-syn (data are means ± SEM, *P < 0.05, ***P < 0.001, and ****P < 0.0001, one-way ANOVA with Tukey’s multiple comparisons test). Bar = 20 μm. AU, Arbitrary unit.

To investigate the effect of lovastatin on α-syn aggregation, we treated the α-syn-GFP-HEK293 cells with different concentrations of lovastatin for 4 h, and then transduced with α-syn PFFs. 48 h later, the cells developed globular and thread-like inclusions. The number of intracellular aggregates was reduced by lovastatin in a concentration-dependent manner (Figures 1A,C). However, the amount of α-syn PFFs entering the cells was not altered (Supplementary Figure 1), indicating that lovastatin does not affect the uptake of α-syn PPFs. After permeabilization, soluble α-syn leak into the outside of the cells, while insoluble α-syn remained. Lovastatin decreased the density of insoluble α-syn in the cells in a concentration-dependent manner (Figures 1B,D). Overall, these observations indicate that lovastatin inhibits α-syn aggregation induced by α-syn PFFs.

### Lovastatin Attenuates α-Syn Phosphorylation in α-Syn-GFP-HEK293 Cells

**FIGURE 2:**
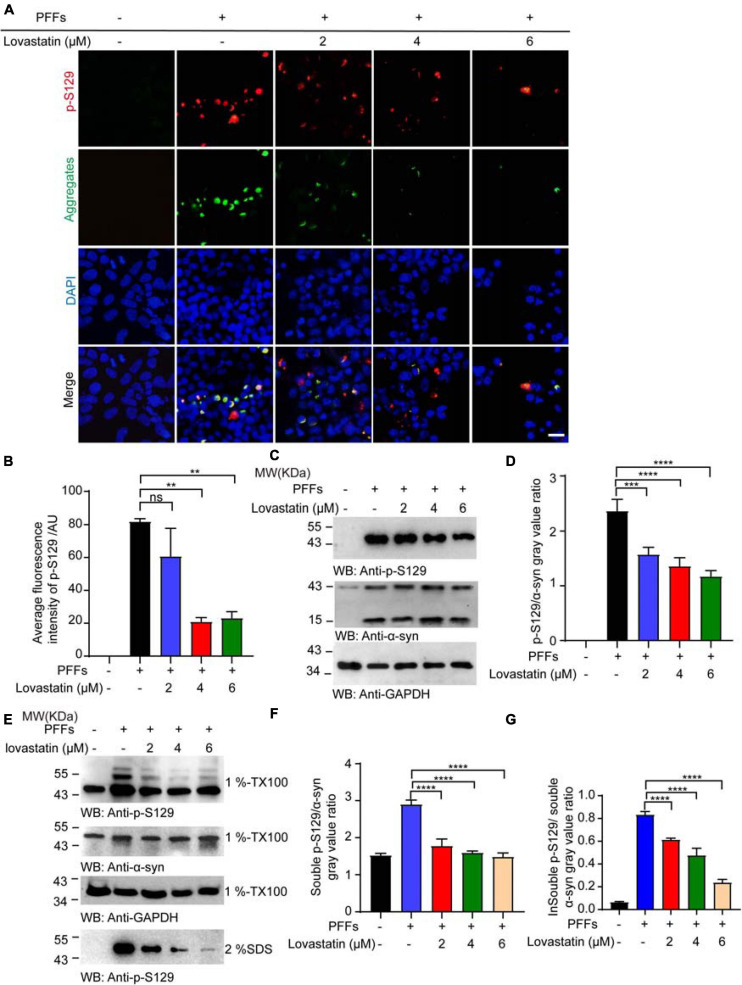
Lovastatin attenuates α-syn phosphorylation in GFP-α-syn-HEK293 cells. **(A)** Immunofluorescence showing that p-S129 co-localized with α-syn aggregates. Intracellular p-S129 α-syn (red) was decreased in the lovastatin group. **(B)** Quantitative analysis of p-S129 α-syn fluorescence. **(C)** Western blot analysis of the p-S129 level. **(D)** Quantification of the p-S129 level in Western blot. Results are normalized to total α-syn. **(E)** Western blot analysis of soluble and insoluble p-S129 α-syn. **(F)** Quantification of soluble p-S129 α-syn. Results are normalized to total α-syn. **(G)** Quantification of insoluble p-S129 α-syn. Results are normalized to total α-syn. All data are means ± SEM, **P < 0.01, ***P < 0.001, and ****P < 0.0001, ns: not statistically significant, one-way ANOVA with Tukey’s multiple comparisons test. Bar = 20 μm. All experiments were performed in triplicate for at least three independent times.

Phosphorylation of α-syn at S129 is believed to deeply participate in the onset of PD pathology. To verify the effect of lovastatin on α-syn phosphorylation, the lovastatin-treated α-syn-GFP-HEK293 cells were stained with the anti-p-S129 antibody. It showed that p-S129 colocalized with the GFP-labeled aggregates, suggesting that the insoluble α-syn was phosphorylated at the S129 site (Figure 2A). Compared with the control group, the intracellular p-S129 in the lovastatin-treated group was significantly reduced in a concentration-dependent manner (Figure 2B). Similar results were confirmed by Western blot (Figures 2C,D). We further investigated whether lovastatin is capable of influencing α-syn conformation, and leads to the alteration of the protein solubility. Soluble and insoluble α-syn were extracted from α-syn-GFP-HEK293 cells and analyzed by Western blot. α-Syn PFFs remarkably induced insoluble fractions, which could be attenuated by lovastatin in a concentration-dependent manner (Figures 2E–G). In conclusion, lovastatin relieves PFFs-induced α-syn phosphorylation and aggregation *in vitro*.

### Lovastatin Alleviates the Phosphorylation of Endogenous α-Syn in SH-SY5Y Cells

**FIGURE 3:**
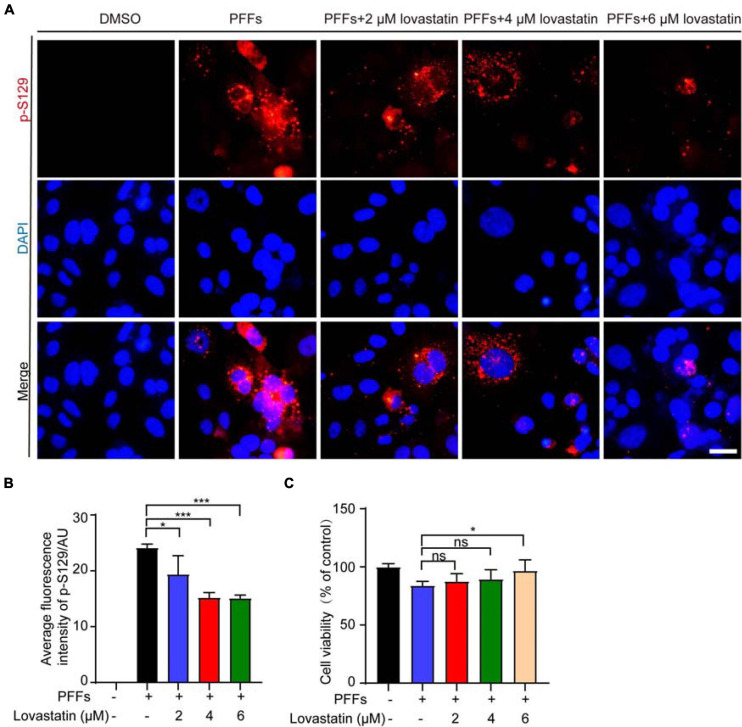
Lovastatin alleviates the phosphorylation of endogenous α-syn in SH-SY5Y cells. **(A)** Immunostaining for p-S129 α-syn showing that lovastatin reduces the phosphorylation of α-syn in SH-SY5Y cells. Bar = 20 μm. **(B)** Quantification of p-S129 α-syn. **(C)** CCK8 analysis showing that 6 μM lovastatin improves cell viability. All data are means ± SEM, *P < 0.05, and ***P < 0.001, ns: not statistically significant, one-way ANOVA with Tukey’s multiple comparisons test. All experiments were performed in triplicate for at least three independent times.

To investigate the effect of lovastatin on the phosphorylation of endogenous α-syn, we treated SH-SY5Y cells with different concentrations of lovastatin for 4 h, and then transfected with α-syn PFFs, with lovastatin maintained in the medium. 48 h later, immunostaining with anti-p-S129 antibody found that lovastatin attenuated the phosphorylation of endogenous α-syn in a concentration-dependent manner (Figures 3A,B). The viability of SH-SY5Y cells was impaired after transfected with α-syn PFFs. Lovastatin partly blocked the toxicity of α-syn PFFs (Figure 3C). Therefore, lovastatin inhibits endogenous α-syn phosphorylation and protects cell viability.

### Lovastatin Inhibits the Activation of CK2

**FIGURE 4:**
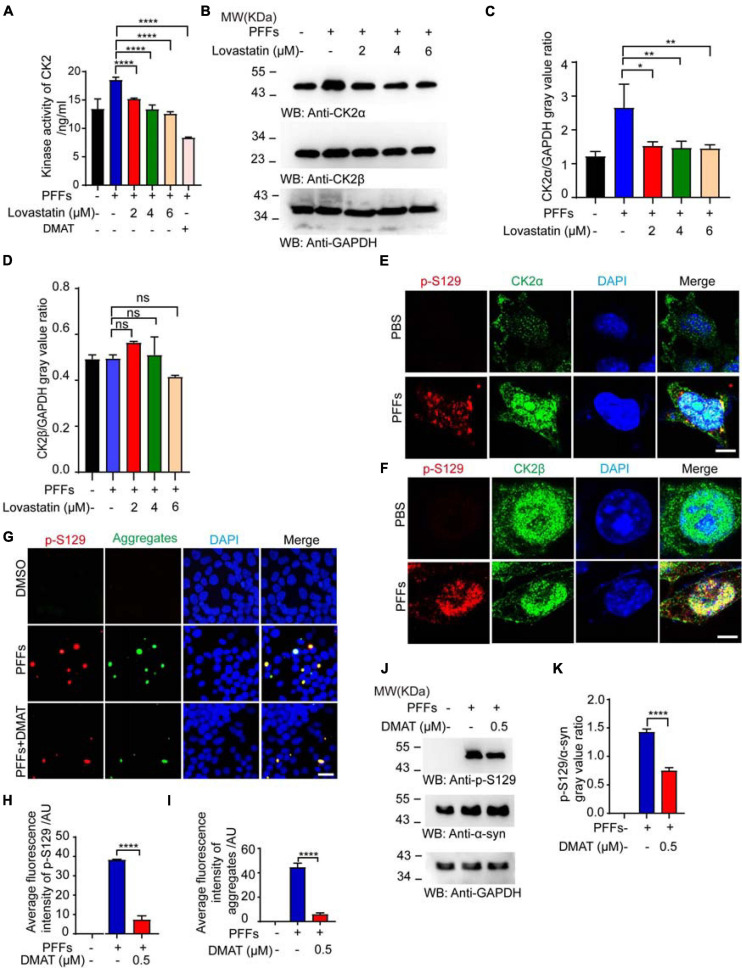
Lovastatin inhibits the activation of CK2. **(A)** Kinase activation of CK2. **(B)** Western blot detecting the levels of CK2α and CK2β. **(C,D)** Quantitative analysis of CK2α and CK2β levels. **(E)** Double-immunostaining for p-S129 and CK2α in the SH-SY5Y treated by α-syn PFFs. Bar = 8 μm. **(F)** Double-immunostaining for p-S129 and CK2β in the SH-SY5Y treated by α-syn PFFs. Bar = 8 μm. **(G)** Immunostaining showing CK2 inhibitor DMAT decreased the phosphorylation and inclusion. **(H)** Quantification of p-S129 α-syn after DMAT treatment. **(I)** Quantification of aggregates after DMAT treatment. **(J)** Western blot detecting the level of p-S129 α-syn. **(K)** Quantification of p-S129 α-syn in Western blot. Results are normalized to total α-syn. All data are means ± SEM, *P < 0.05, **P < 0.01, ***P < 0.001, and ****P < 0.0001, ns: not statistically significant, one-way ANOVA with Tukey’s multiple comparisons test. All experiments were performed in triplicate for at least three independent times.

To explore the mechanisms by which lovastatin reduces α-syn phosphorylation, we assessed the influence of lovastatin on the kinase activity of CK2, which is one of the kinases phosphorylating α-syn (van der Most et al., 2009; Sierra et al., 2011). After the cells were transfected with PFFs, the activation of CK2 was pumped, while lovastatin decreased the activity of CK2 in a concentration-dependent manner (Figure 4A). However, the kinase activity of G protein-coupled receptor kinase 5 (GRK5), one of the other α-syn kinases, was not affected by α-syn PFFs or lovastatin (Supplementary Figure 2). Western blot found that the content of catalytic subunits α increased (Figures 4B–D), but the expression of regulatory subunit β did not change after α-syn PFFs treatment. Furthermore, in α-syn PFFs-treated SH-SY5Y cells, p-S129 colocalized with CK2α and CK2β (Figures 4E,F). To confirm this, we treated the cells with 2-dimethylamino-4,5,6,7-tetrabromobenzimidazole (DMAT), a CK2 inhibitor. DAMT inhibited the PFFs-induced CK2 activation (Figure 4A). DAMT also attenuated the aggregation and phosphorylation of α-syn at S129 in α-syn-GFP-HEK293 cells (Figures 4G–K). These results support that lovastatin decreased the aggregation and phosphorylation of α-syn by inhibiting CK2 activation in these two cell lines.

### Lovastatin Relieves PFFs-Induced Oxidative Stress

**FIGURE 5:**
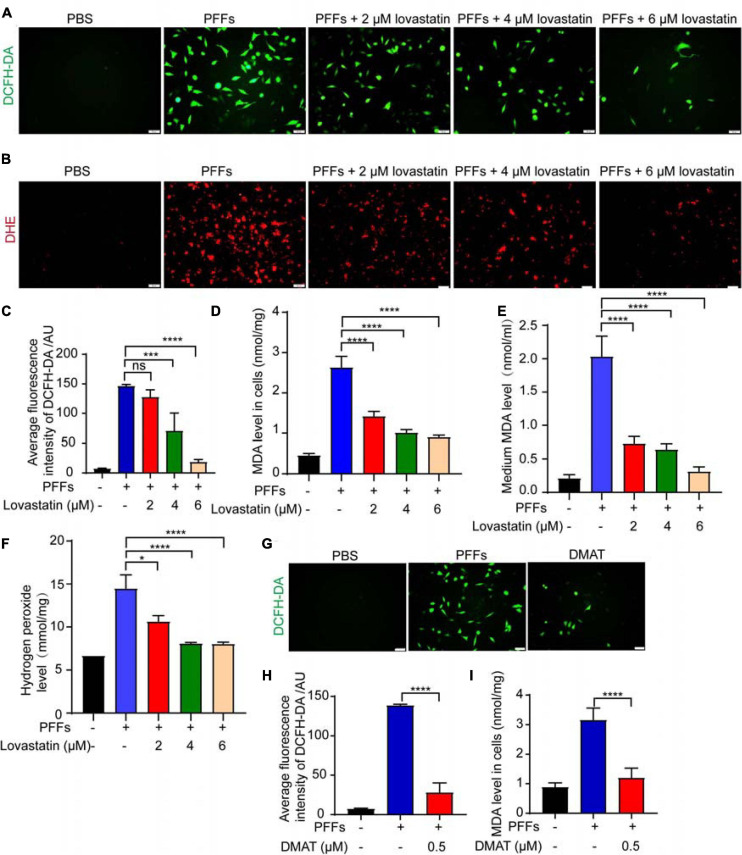
Lovastatin mitigates oxidative stress induced by PFFs. **(A,B)** ROS was detected after lovastatin treatment by DCFH-DA probe **(A)** and DHE probe **(B)**. Bar = 50 μm. **(C)** Quantification of the DCFH-DA florescence. **(D)** Quantification of MDA after lovastatin treatment in the SH-SY5Y cells. **(E)** Quantification of MDA after lovastatin treatment in the medium. **(F)** Quantification of hydrogen peroxide in the SH-SY5Y cells. **(G)** ROS was detected after DMAT treatment by DCFH-DA probe. Bar = 50 μm. **(H)** Quantification of DCFH-DA florescence after DMAT treatment. **(I)** Quantification of cellular MDA after DMAT treatment. All data are means ± SEM, *P < 0.05, and ****P < 0.001, ns: not statistically significant, one-way ANOVA with Tukey’s multiple comparisons test. All experiments were performed in triplicate for at least three independent times.

It has been reported that α-syn PFFs induce oxidative stress and promote neuronal death in PD (Musgrove et al., 2019). Thus, we further examined the effect of lovastatin on PFFs-induced oxidative stress using SH-SY5Y cell line. The levels of ROS, MDA and hydrogen peroxide were increased after α-syn PFFs administration, while lovastatin concentration-dependently reduced the levels of superoxide anion (Figures 5A–C). It also reduced the levels of MDA and hydrogen peroxide induced by PFFs (Figures 5D–F). We also tested whether CK2 contributed to the PFFs-induced oxidative stress in SH-SY5Y cells. CK2 inhibitor, DMAT, significantly decreased ROS levels when compared with the control group (Figures 5G–I). Together, these findings suggest that lovastatin relieves oxidative stress induced by α-syn PFFs in SH-SY5Y cells.

### Lovastatin Downregulates PFFs-Induced Histone Acetylation

**FIGURE 6:**
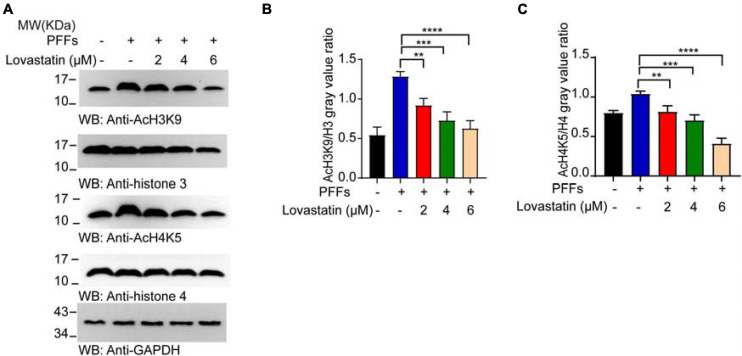
Lovastatin mitigates the acetylation of the histone induced by PFFs. **(A)** Western blot detecting the content of AcH4K5, AcH3K9, Histone 3, and Histone 4. **(B)** Quantification of AcH3K9. Results are normalized to Histone 3. **(C)** Quantification of AcH4K5. Results are normalized to Histone 4. All data are means ± SEM, *P < 0.05, **P < 0.01, ***P < 0.001, and ****P < 0.001, one-way ANOVA with Tukey’s multiple comparisons test. All experiments were performed in triplicate for at least three independent times.

Increased histone acetylation has been reported in the dopaminergic neurons of animal models and PD patients (Monti et al., 2010). Abnormal histone acetylation such as histone H4 lysine 5 acetylation (AcH4K5) and H3 lysine 9 acetylation (AcH3K9) contribute to the pathogenesis of PD (Park et al., 2016). We examined the effect of α-syn PFFs on histone acetylation in the α-syn-GFP-HEK293 cell line. It suggests that α-syn PFFs increased the expression of AcH4K5 and AcH3K9 and both could be decreased by lovastatin treatment (Figures 6A–C). In conclusion, lovastatin attenuates PD-related histone acetylation in α-syn-GFP-HEK293 cells.

## Discussion

In this study, we described that the cholesterol-lowering drug lovastatin attenuates the phosphorylation and aggregation of α-syn in cellular models of PD. Lovastatin decreases the expression of CK2 and inhibits its kinase activity. Besides, lovastatin also attenuated oxidative stress and epigenetic dysregulation induced by α-syn PFFs. Our findings provide evidence supporting the protective effects of lovastatin in the development of PD pathology (Lin et al., 2021), and shed light on applying the conventional cholesterol-lowering drug to PD management.

Parkinson’s disease is the most common synucleinopathy, which is characterized by the aberrant aggregation of α-syn in the central nervous system (Homayoun, 2018). It occupies the fastest growing prevalence, disability, and mortality rate among all neurological disorders, which has been bringing a huge economic burden worldwide (Marinho de Souza et al., 2018). Normally α-syn is expressed in the presynaptic terminals of neurons, and exists in the form of soluble monomers. However, under pathological conditions, the S129 site of α-syn is aberrantly phosphorylated, which alters its secondary structures and promotes them to assemble into β-sheet-rich aggregates, stimulating a series of negative biochemical processes such as oxidative stress, inflammation, mitochondrial dysfunction, etc. It eventually leads to the degeneration of dopaminergic neurons and the onset of PD.

Recent reports have shown that the phosphorylation and aggregation of α-syn are closely related to lipid droplets. Abnormal lipids promote the misfolding of α-syn. Statins might decrease the incidence of PD by restraining the aggregation of α-syn. Clinical evidence has shown that lovastatin demonstrated the most significant effect (Wahner et al., 2008). However, the molecular mechanisms have not been explored yet. In this study, we characterized the effects of lovastatin on the α-syn phosphorylation and aggregation using two cellular models. Compared to neurotoxin-induced cellular models, the advantage of these two cellular models is that they manifest stable and remarkable α-syn accumulation and phosphorylation. We found that insoluble α-syn aggregates appeared after treated with PFFs. Furthermore, the insoluble α-syn colocalized with p-S129, which well-mimicked the similar pathological features in PD patients. In the presence of lovastatin, the phosphorylation and aggregation of intracellular α-syn were significantly reduced.

Casein kinase 2 is one of the kinases responsible for the phosphorylation of α-syn (Perez et al., 2011). α-Syn PFFs significantly activated the kinase property of CK2, accompanied by the increased expression of its catalytic subunit α. This arose an explanation for PFFs-induced endogenous α-syn phosphorylation. In addition, lovastatin decreased PFFs-induced oxidative stress and epigenetic dysregulation in SH-SY5Y cells, indicating that lovastatin may exert protective effects via several pathways. We found that lovastatin reduced the aggregation of α-syn, which is consistent with the previous report by Bar-On et al. (Bar-On et al., 2008). However, they found that lovastatin did not affect the phosphorylation of α-syn. This might be ascribed to the distinct cellular models that we adopted. Bar-On et al. used B103 rat neuroblastoma cells stably expressing α-syn in the absence of any additional treatments for promoting α-syn aggregation and phosphorylation. Since most of the S129 sites in the α-syn aggregates in the brains of PD patients are phosphorylated, p-S129 is widely used as a biomarker for assessing α-syn aggregation. In the models we used, treatment with α-syn PFFs significantly aggravated intracellular α-syn aggregation, phosphorylation, and oxidative stress. Meanwhile, the phosphorylation level was highly consistent with the degree of aggregation. Thus, the PFFs-induced α-syn aggregation models are more consistent with PD. Our results are in agreement with the observation that lovastatin attenuates α-synuclein aggregation in transgenic mouse models overexpressing human α-syn. Besides, lovastatin has been found to inhibit the oxidation of α-syn (Koob et al., 2010). The effect of lovastatin on oxidative stress may result from reduced cholesterol levels. However, statins may also perform antioxidant effects independent from their cholesterol-lowering effect, such as inhibition of oxidative stress or modulation of neuroinflammation (van der Most et al., 2009; Sierra et al., 2011; Liu et al., 2021).

Recently, a double-blind, randomized, placebo-controlled trial including 77 patients with early-stage PD showed that administration of high-dose lovastatin in early-stage PD patients has a trend toward decreasing motor symptom progression and slowing the dopaminergic neuronal decline (Lin et al., 2021). Besides, it has been reported that a large proportion of individuals with recent-onset PD have increased cardiovascular risk, which is associated with greater motor and cognitive severity, and greater axial impairment. Administration of statins in PD patients with increased vascular risk could reduce cardiovascular events and thereby slowed the progression of motor and cognitive decline (Swallow et al., 2016). Thus, the effect of lovastatin on α-syn phosphorylation, oxidative stress, the level of cholesterol, and histone acetylation may contribute to its protective effect on PD.

Epigenetics, the process of changing gene activity without changing genetic information, is thought to contribute to neuronal cell death in PD (Harrison and Dexter, 2013). One of the most intensively studied modes of epigenetic regulation is the post-translational modification of the N-terminal tails of histone, around which DNA is normally coiled (Labbé et al., 2016). Higher levels of histone acetylation are found in the midbrain of dopaminergic neurons from PD patients compared to controls (Park et al., 2016). A recent study has shown that the dopaminergic neurotoxin 1-methyl-4-phenylpyridinium (MPP^+^) increases the levels of acetylated histones in experimental models of PD (Park et al., 2016). Furthermore, paraquat and dieldrin also induced the hyperacetylation of core histones H3 and H4 and resulted in apoptosis of dopaminergic neurons (Song et al., 2010). 6-hydroxydopamine (6-OHDA) increased histone acetylation by decreasing the activity of tubulin deacetylase SIRT2 (Patel and Chu, 2014). In addition, several PD-related genes are regulated by histone modifications. For example, α-syn is regulated by histone acetylation and it can also regulate histone acetylation in turn. *In vitro*, histones interact with α-syn in the nucleus and accelerate α-syn fibrillation and toxicity (Goers et al., 2003). These evidence indicates that regulating histone acetylation may be one of the ways to treat PD. In our study, we found that α-syn PFFs increased the level of AcH4K5 and AcH3K9, which was attenuated by lovastatin treatment. In addition to the effect of statins on histone modification, statin users had significantly lower DNA methylation levels than the control group in a blood epigenome association study (Ochoa-Rosales et al., 2020). It is reported that the methylation of the two genes involved in cholesterol synthesis, 24-dehydrocholesterol reductase (DHCR24) and ATP binding cassette subfamily G member 1 (ABCG1), was significantly reduced in subjects on statin therapy (Ochoa-Rosales et al., 2020; Schrader et al., 2021). These evidence indicates that statins may exert protective effect by attenuating epigenetics dysregulation in PD.

In summary, lovastatin attenuates α-syn PFFs-induced phosphorylation and aggregation of α-syn, decreased oxidative stress, attenuated histone acetylation, and protected cell viability. These results provide new insights into the mechanisms by which lovastatin reduces the risk of PD.

## Data Availability Statement

The original contributions presented in the study are included in the article/Supplementary Material, further inquiries can be directed to the corresponding author.

## Author Contributions

ZZ conceived the project and designed the experiments. LD performed most of the experiments and wrote the original draft. JW helped with the cell culture. MH, MX, YT, and CL helped in the data analysis. All authors contributed to the article and approved the submitted version.

## Conflict of Interest

The authors declare that the research was conducted in the absence of any commercial or financial relationships that could be construed as a potential conflict of interest.

## Publisher’s Note

All claims expressed in this article are solely those of the authors and do not necessarily represent those of their affiliated organizations, or those of the publisher, the editors and the reviewers. Any product that may be evaluated in this article, or claim that may be made by its manufacturer, is not guaranteed or endorsed by the publisher.

## Supplementary Material

Supplementary Figure 1 **(A)** Immunofluorescence showing the presence of exogenous his-α-syn PFFs in cells. **(B)** Quantification of his-α-syn PFFs in cells. All data are means ± SEM, ns: not statistically significant, one-way ANOVA with Tukey’s multiple comparisons test. All experiments were performed in triplicate for at least three independent times.

Supplementary Figure 2 Quantitative detection of GRK5 activity. All data are means ± SEM, ns: not statistically significant, one-way ANOVA with Tukey’s multiple comparisons test. All experiments were performed in triplicate for at least three independent times.

## Abbreviations

PD: Parkinson’s disease
SNpc: substantia nigra pars compacta
CK2: casein kinase 2
PFFs: pre-formed fibers
Lewy bodies: LBs
ROS: reactive oxygen species
AcH4K5: histone H4 lysine 5 acetylation
AcH3K9: histone H3 lysine 9 acetylation
DMAT: 2-dimethylamino-4,5,6,7-tetrabromobenzimidazole.
