# Supplementary figures and images for: Lovastatin Alleviates α-Synuclein Aggregation and Phosphorylation in Cellular Models of Synucleinopathy
Source: Front Mol Neurosci. 2021 Jul 26;14:682320. doi: 10.3389/fnmol.2021.682320 (PMC8350347; doi:10.3389/fnmol.2021.682320)

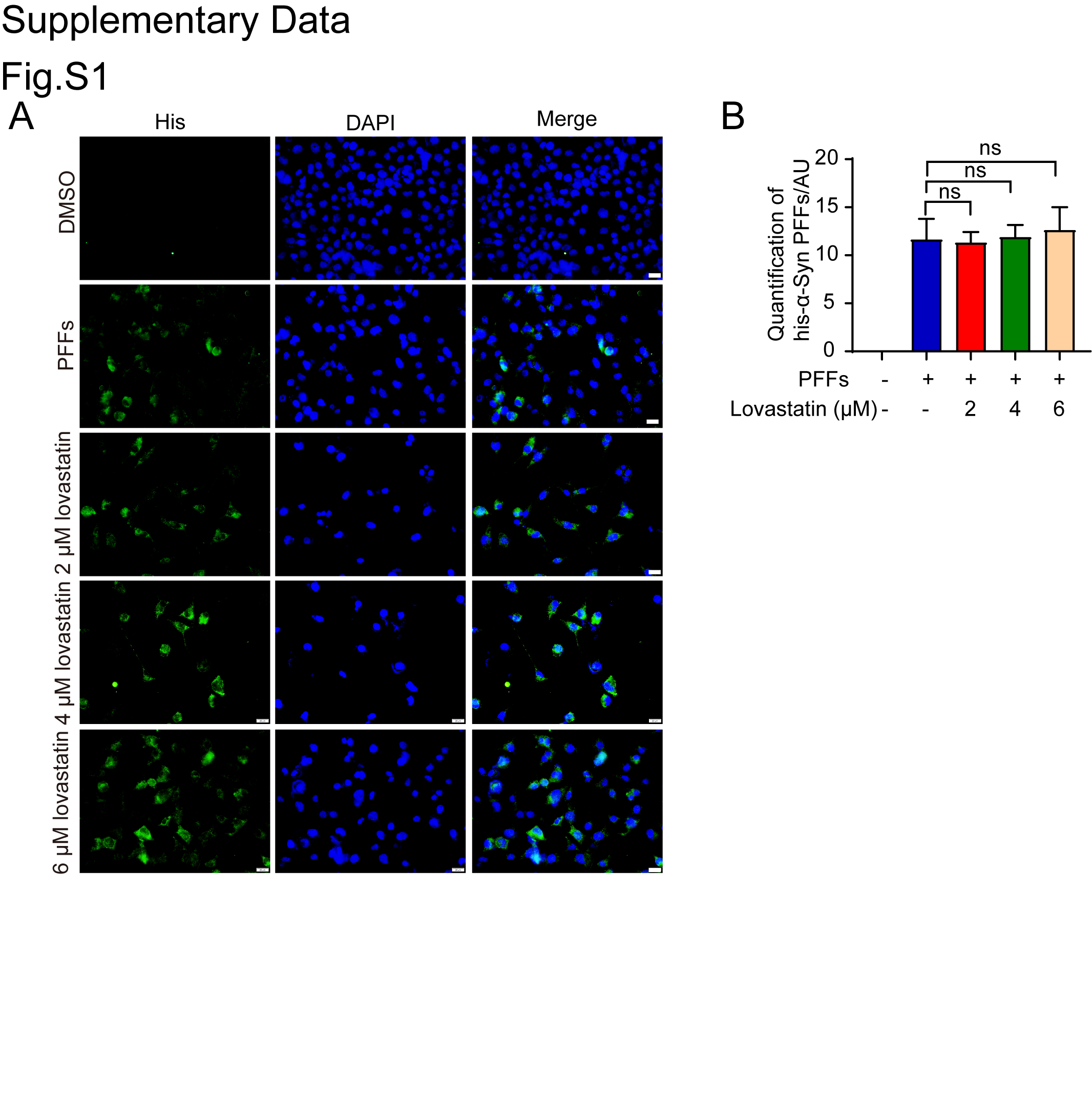

Supplement: Supplementary Figure 1 — (A) Immunofluorescence showing the presence of exogenous his-α-syn PFFs in cells. (B) Quantification of his-α-syn PFFs in cells. All data are means ± SEM, ns: not statistically significant, one-way ANOVA with Tukey’s multiple comparisons test. All experiments were performed in triplicate for at least three independent times. [file Image_1.TIF]

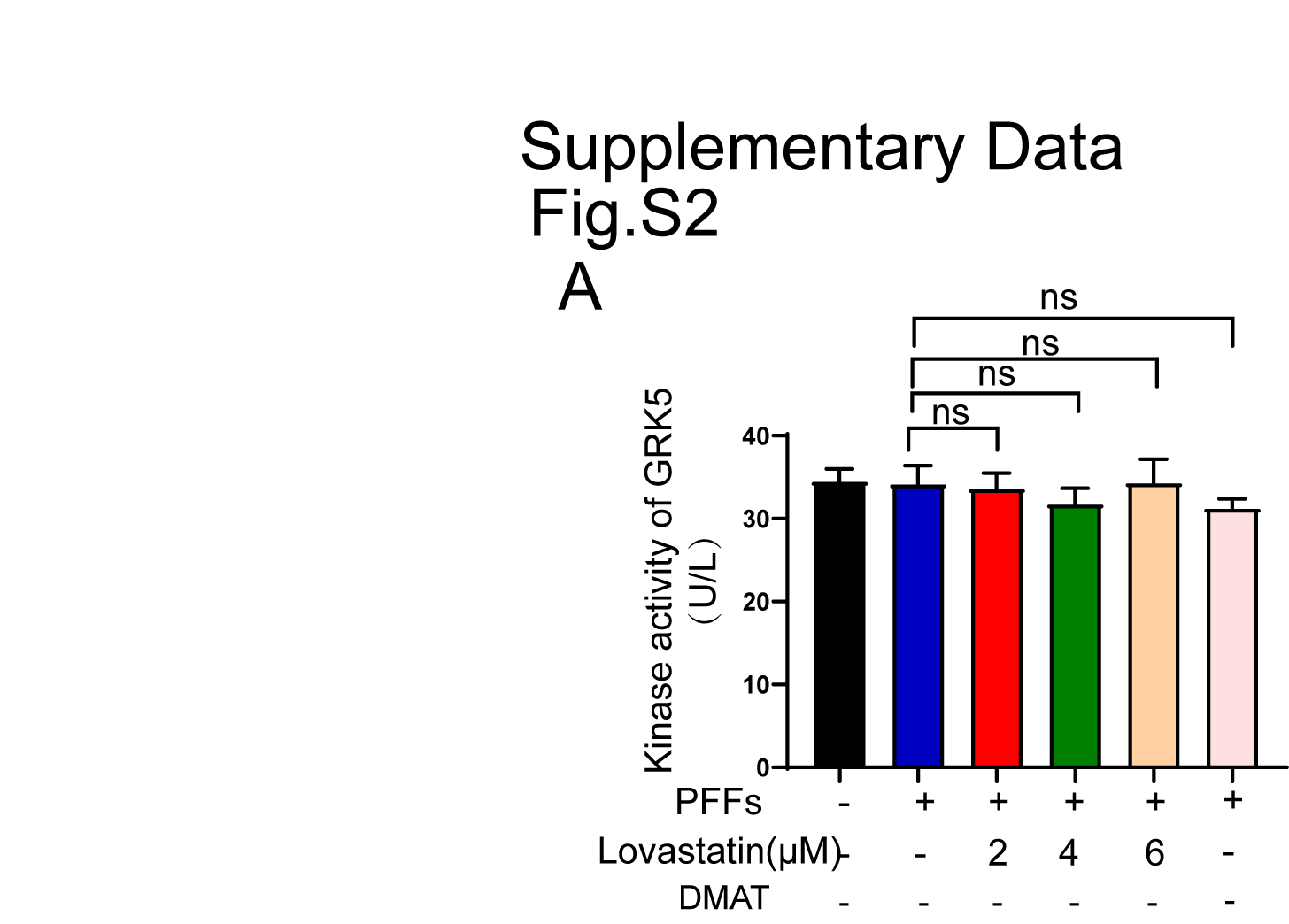

Supplement: Supplementary Figure 2 — Quantitative detection of GRK5 activity. All data are means ± SEM, ns: not statistically significant, one-way ANOVA with Tukey’s multiple comparisons test. All experiments were performed in triplicate for at least three independent times. [file Image_2.TIF]
